# UEGJ—The Journey has Only Just Begun

**DOI:** 10.1002/ueg2.70080

**Published:** 2025-08-13

**Authors:** Albrecht Neesse

**Affiliations:** ^1^ Department of Gastroenterology Gastrointestinal Oncology and Endocrinology University Medical Center Goettingen Goettingen Germany

This issue represents a compilation of recently published articles in the UEG Journal that we would like to share with you during your visit to UEGW in Berlin in a special print format. The articles were carefully selected by our Editorial team to showcase the quality and breadth of the journal including this year's UEGJ best paper award winner [1]. I hope you will find time during the next few busy days in Berlin or during your trip back home to scroll through this exciting collection.

Personally, it is an immense honour that I represent UEG Journal at UEGW 2025 for the first time as new Editor in Chief. When I took the helm from Joost PH Drenth in January 2025, the journal could not have been in a better shape [2]. The transition was smooth and started several months earlier, and I am immensely grateful for all the hard and successful work that was done by Joost and his team of Associate and Trainee Editors. In particular, I would like to thank the outgoing Associate Editors Alexander Meining, Fernando Magro, Christopher Moreno and Mao Ren for their enthusiasm, expertise and daily dedication that shaped the high‐quality format of the journal, and I continue to ask them for their valuable advice when difficult decisions have to be made.

UEG Journal was launched in 2013 and can still be considered a young journal within the highly competitive publishing landscape. However, UEG Journal has rapidly become one of the leading clinical and translational Gastroenterology journals that has seen a remarkable development in recent years and has become a first‐class, high‐impact platform for clinical and translational studies covering all areas of gastroenterology, hepatopancreaticobiliary diseases [3], inflammatory bowel diseases [4], neurogastroenterology, endoscopy [5], and GI oncology. In addition, a growing number of consensus guidelines have been published in UEG Journal that we offer to you in conjunction with UEG's quality and care committee and other GI societies [6, 7].

The number of submitted manuscripts has steadily increased over the last years meaning that we can now select the most exciting and impactful findings to be published in UEGJ. As a result, our new impact factor for 2024 is 6.7 moving us amongst the top 12% of all GI journals and well within our initial goal to position ourselves within the best 15% of GI journals. On this occasion, I would like to take the opportunity to thank all parties involved, past and current Associate and Trainee Editors, numerous peer‐reviewers, authors, readers, the two past Editors in Chief, Jan Tack and Joost PH Drenth, our publisher Wiley and United European Gastroenterology (UEG).

Looking ahead, the journey of UEG journal has only just begun and will continue with a fantastic group of professionals. We have a dedicated and vibrant team of Associate Editors with Daniel Keszthelyi (Maastricht, Netherlands), Yasuko Maeda (Glasgow, UK), and newly appointed Associate Editors Konrad Aden (Kiel, Germany), Andrea Anderloni (Pavia, Italy), Elisa Pose (Barcelona, Spain), Keith Siau (Cornwall, UK) and Robert Verdonk (Nieuwegein, Netherlands). Each of them brings special expertise in their respective fields to the table, and we are in continuous exchange and scientific debate (in person, virtually, via phone) to select the best and most impactful papers for our readers. In addition, Katharina Buder‐Gannon (Head of Marketing & Communication) and Andrea Nowak (Governance & Community Management) from UEG Headquarters in Vienna support my work as Editor in Chief on a daily basis. Our joint goal is to maintain and further strengthen the excellent reputation and high‐quality of UEG Journal in the coming years.

However, UEG Journal is much more than just another high‐impact journal. In 2025, we selected, with the help of the UEG Young Talent Group, the third round of 14 Trainee Editors for the journal. These highly‐talented clinicians and scientists are from France, Spain, Portugal, Italy, Netherlands, Germany, UK, Slovenia, Romania and Finland and join the Editorial Board for 2 years. The UEG Journal values the combination of Senior and Trainee Editors and is committed to values of diversity and inclusion [8]. The Trainee Editors are an essential part of UEG Journal providing us with a constant flow of fresh perspectives, ideas and enthusiasm. All Trainee Editors significantly contribute to the success of the journal by being involved at various stages of the publishing process such as peer‐reviewing, visual‐abstract creation, social media outreach, search‐engine optimization or podcast recording. Senior and Trainee Editors regularly meet up in person or virtually to discuss future strategies and learn from each other thus building a strong professional and educational network within UEG Journal and beyond.

In the coming years, the Editorial team and the publisher Wiley will work tirelessly to further improve the service for our submitting authors. Confidentiality, speed and constructive criticism are key pillars for a successful author‐editor relationship including rapid online visibility upon acceptance of the manuscript. The Board of Editors looks at each submitted manuscript very carefully and evaluates its scientific quality, novelty and impact in the respective field. With the help of our expert peer‐reviewers, our aspiration is to improve the submitted manuscript in all aspects. In case of acceptance, UEG Journal will provide support in design, visual abstract creation and promotion of the manuscript via our social media channels to spread the message of your high‐quality science.

Importantly, UEG Journal is the flagship of UEG, a growing community of over 50.000 professionals dedicated to advancing the prevention and care of digestive diseases in Europe and beyond. UEGW 2025 is yet another example of the global growth, impact and innovative nature of UEG that has become one of the largest and most influential Gastroenterology societies world‐wide with a growing number of scientific, educational, and social activities. The close collaboration with UEG is a major asset for the journal.

Let's continue this exciting and promising journey together!

I hope you enjoy UEGW 2025 in Berlin, and I will see you next year in Barcelona.

## Conflicts of Interest

The author declares no conflicts of interest.

## Data Availability

Data sharing not applicable to this article as no datasets were generated or analysed during the current study.
